# Assessing the utility of virtual OSCE sessions as an educational tool: a national pilot study

**DOI:** 10.1186/s12909-022-03248-3

**Published:** 2022-03-15

**Authors:** Sarika Grover, Maharsh Pandya, Chavini Ranasinghe, Saajan P. Ramji, Harroop Bola, Siddarth Raj

**Affiliations:** 1 GKT School of Medicine, King’s College London, London, UK; 2 Centre for Medical Education, School of Medicine, Cardiff University, Cardiff, UK; 3 University College London Medical School, University College London, London, UK; 4 Imperial College School of Medicine, Imperial College London, London, UK

**Keywords:** Virtual, Objective structured clinical examination (OSCE), Educational tool, National pilot study

## Abstract

**Background:**

Objective Structured Clinical Examinations (OSCEs) are a common form of assessment used across medical schools in the UK to assess clinical competence and practical skills and are traditionally held in an in-person format. In the past, medical students have often prepared for such exams through in-person peer-assisted learning (PAL), however, due to the recent Covid-19 pandemic, many in-person teaching sessions transitioned to online-based formats. There is currently a paucity of research on the utility of virtual PAL OSCE sessions and thus, we carried out a national pilot study to determine the feasibility of virtual OSCE teaching via feedback from participants and examiners.

**Methods:**

A total of 85 students from 19 UK-based medical schools with eight students based internationally attended the series of online OSCE workshops delivered via Zoom®. All students and examiners completed a feedback questionnaire at the end of each session regarding parameters, which included questions on pre-and post-workshop confidence in three OSCE domains: history-taking, communication and data interpretation. A Likert scale using 5 Likert items was used to self-report confidence, and the results were analysed using the Mann-Whitney U test after assessing for normality using the Shapiro-Wilk test.

**Results:**

Results from student feedback showed an increase in confidence for all three OSCE domains after each event (*p* < 0.001) with 69.4% agreeing or strongly agreeing that online OSCE sessions could sufficiently prepare them for in-person exams. Questionnaire feedback revealed that 97.6% of students and 86.7% of examiners agreed that virtual OSCE teaching would be useful for preparing for in-person OSCE examinations after the pandemic.

**Conclusion:**

Most participants in the virtual OSCE sessions reported an improvement in their confidence in history-taking, communication and data interpretation skills. Of the participants and examiners that had also experienced in-person OSCE examinations, the majority also reported that they found virtual OSCE sessions to be as engaging and as interactive as in-person teaching. This study has demonstrated that virtual OSCE workshops are a feasible option with the potential to be beneficial beyond the pandemic. However, more studies are required to assess the overall impact on student learning and to determine the value of virtual OSCE workshops on exam performance.

**Supplementary Information:**

The online version contains supplementary material available at 10.1186/s12909-022-03248-3.

## Introduction

Objective structured clinical examinations (OSCEs), since being introduced in 1975, have become a widely recognised form of assessment across medical schools in the United Kingdom (UK) [1]. This in-person examination requires students to rotate between a series of stations, each of which usually lasts between six to 15 min [2, 3]. Each station typically assesses a core skill or combination of skills that include, but are not limited to, simulated consultations, clinical examinations, practical procedures and clinical data interpretation [2, 3].

Clinical teaching in the format of peer-assisted learning (PAL) is a common form of OSCE preparation [4]. Two separate studies assessing the effectiveness of in-person PAL OSCE teaching by Rashid et al. and Bevan et al. concluded that in-person PAL OSCE teaching was not only useful for all participants but significantly improved participant confidence with students stating that they felt ‘better equipped’ for their OSCEs [5, 6].

The recent Coronavirus (COVID-19) pandemic has severely restricted the ability to organise and deliver in-person teaching, and many aspects of medical education have been forced to shift to online learning via platforms such as Zoom® as a way to reduce social contact [7, 8]. Given the importance placed on OSCEs by medical schools, along with the fact that in-person OSCE teaching was not possible due to the ongoing pandemic, virtual sessions have served as a potentially viable alternative for delivering OSCE teaching.

One study by Prettyman et al. that involved the delivery of virtual OSCE teaching for nurse practitioner students in America stated that some key advantages in comparison to in-person teaching included: ease of attendance despite physical distance, practising and teaching relevant skills for virtual consultations for future virtual clinics and the ease of setup in comparison to in-person teaching [9].

Currently, there is a scarcity of research on the effectiveness of virtual OSCE teaching for medical students. Existing studies in the UK mainly focus on virtual OSCE teaching specific to a given medical school [4]. Therefore, while there is emerging literature on the trial and delivery of virtual OSCE teaching, there is still a paucity of evidence regarding the advantages and disadvantages in comparison to in-person OSCE teaching, specific to all medical students nationwide [10, 11].

During the height of the COVID-19 pandemic, a few medical schools opted to deliver final year OSCEs in a virtual format, while some choose to postpone or cancel them altogether [12]. However, with the UK easing COVID-19 restrictions, it is likely that in-person OSCE examinations are bound to resume nationwide, and hence we must reconsider whether virtual OSCE teaching has a role to play once the necessity for virtual teaching and examining diminishes.

Therefore, this pilot study aims to use post-intervention participant feedback to assess the feasibility of a nationally delivered virtual OSCE workshop by understanding participant confidence in the following domains: history taking, communication and data interpretation before and after the workshop. This study will look at factors such as engagement and interactivity of virtual OSCE workshops in comparison to in-person teaching and therefore identify the scope for future virtual OSCE teaching in a post-pandemic landscape.

## Methods

The methods in this study were performed following the STROBE guidelines for reporting observational studies, which include a checklist for cross-sectional studies [13].

### Workshops

The national OSCE teaching was delivered as a series of online workshops delivered on a weekly basis via Zoom® (Zoom Video Communications, USA), from February to March 2021 as a nationally organised student-led initiative. It was identified that there was a deficit in the availability of online structured OSCE teaching that supported the student experience with both practice and detailed feedback. Therefore, launching an OSCE workshop series was an appropriate medium to widen accessibility and utility to improve clinical practice. The sessions focused on practising common OSCE stations, including history-taking, communication and data interpretation skills. Sessions were delivered for each of the commonly examined specialities: cardiology, respiratory, gastroenterology, neurology and endocrinology.

In total, the series was composed of five consecutive workshops, attended by 85 students distributed throughout. Each session had a total of six OSCE stations and consisted of two circuits with three stations per circuit. A single circuit composed of one history-taking station, one communication station and one data interpretation station, each of which was overseen by an assigned examiner. The examiners were clinical-year medical students at various medical schools across the UK and they led, facilitated and delivered feedback for their assigned station. The maximum number of students in each session was limited to 36 in order to ensure that the examiners were able to provide detailed and individualised feedback within the allocated time. The study was open to all students currently enrolled in a medical school, at any stage, either in the UK or abroad. Students voluntarily signed up for the workshops via an online application form, advertised through social media platforms, and were allocated to each session on a first-come, first-served basis.

The format remained the same for both the history-taking and communication stations. Resources were prepared in advance by the examiners and students were assigned pairs within their groups of six; each pair then took it in turn to practice a scenario. One student would take on the role of the patient while the other would serve as the active participant. The role of the student would alternate between the communication and history-taking stations, thereby giving students the opportunity to both practice skills and contribute to the feedback for their peers. Simultaneously, the examiners would review participant performance during the stations and provide individualised feedback, thereby opening space for reflection. At the end of each session, the examiners would collectively go over the predesigned mark scheme with each group of six with the aim of exploring how to approach a similar station. The data interpretation station was delivered in a group-teaching format, whereby the examiners discussed scenarios with the students and offered methods, advice and tips on tackling a similar station in the exam.

### Student feedback

At the end of each workshop, each of the students was given a feedback questionnaire which asked those who had received previous in-person OSCE teaching, to state how strongly they agreed with the following: online OSCE teaching is as interactive, as engaging, as useful in enabling to them develop relevant clinical skills and providing constructive and helpful feedback. The questionnaire also asked all of the students to rank their confidence in history-taking, communication and data interpretation for that speciality before and after the session, which was graded on a 5-point range of Likert items where 1 = not confident at all and 5 = very confident. These items were chosen to quantify students’ perceived confidence. Importantly, students were also asked whether they thought that online OSCE sessions would be useful in the post-pandemic phase.

### Examiner feedback

The examiners were given similar questionnaires asking them to state how strongly they agreed or disagreed with the same comparative statements regarding online and in-person OSCE teaching, and whether they felt that online-based OSCE teaching would be useful for learning after the pandemic.

### Statistical methods

Attendees completed post-workshop questionnaires and data was collected regarding the demographics of the students and feedback of the workshops. Five-point Likert items were used to quantify the qualitative opinions and perceptions of students and examiners. The Shapiro-Wilk test was used to assess whether the data was normally distributed, and the Mann-Whitney U test was used to assess statistical differences between nonparametric pre- and post-event responses.

### Ethics

This study involved no patients and consisted entirely of both preclinical and clinical medical students attending an undergraduate student-led workshop, requiring no medical or personal information. All participants in this study gave approval for their anonymous data to be used towards potential future research.

## Results

### Students

A total of 85 students attended the five sessions with an average of 17 (range 10-22) students per session, of these students, 64 identified as female and 21 as male. These students included 57 (67.1%) clinical medical students and 28 (32.9%) pre-clinical medical students across 19 UK-based medical schools, with eight students attending from medical schools outside of the UK. This is demonstrated in Table 1.

**Table 1:** Baseline characteristics of participants attending the five virtual OSCE sessions

Parameter	*N* = 85
*Sex*	
Female	64 (75.3%)
Male	21 (24.7%)
Prefer not to say	0 (0%)
*Stage of Training*	
Pre-clinical	28 (32.9%)
Clinical	56 (65.9%)
Intercalating	1 (0.01%)
*Location of medical school*	
UK-based	77 (90.6%)
International	8 (0.10%)
*Received previous in-person OSCE teaching?*	
Yes	59 (69.4%)
No	26 (30.6%)

Students were asked if they had previously received in-person OSCE teaching at any point prior to the virtual event, to which 59 (69.4%) of students stated that they had. Of those who had received in-person OSCE teaching, 48 (81.4%) agreed or strongly agreed that virtual OSCE teaching was as engaging, 49 (83.1%) agreed or strongly agreed that it was as interactive and 48 (81.4%) agreed or strongly agreed that it enabled them to further develop and enhance their previous history-taking, communication and data interpretation skills. Finally, 52 (88.1%) students agreed or strongly agreed that OSCE teaching provided them with appropriate feedback on their performance for preparation of medical school OSCEs. This is shown in Table 2.

**Table 2:** Frequency count of each Likert item as answered by participants on the topic of online versus in-person OSCE teaching (*n* = 59)

	Strongly Disagree (Likert item 1)	Disagree (Likert item 2)	Neutral (Likert item 3)	Agree (Likert item 4)	Strongly Agree (Likert item 5)
Online OSCE teaching is as engaging	1 (1.7%)	3 (5.1%)	7 (11.9%)	32 (54.2%)	16 (27.1%)
Online OSCE teaching is as interactive	1 (1.7%)	1 (1.7%)	8 (13.6%)	34 (57.6%)	15 (25.4%)
Online OSCE teaching enables me to develop clinical skills	2 (3.4%)	5 (8.4%)	4 (6.8%)	31 (52.5%)	17 (28.8%)
Online OSCE teaching provides me with appropriate feedback of my performance	1 (1.7%)	1 (1.7%)	5 (8.4%)	31 (52.5%)	21 (35.6%)

Out of the total 85 participants, most students (69.4%) agreed or strongly agreed that online OSCE sessions could sufficiently prepare them for in-person OSCE examinations, and 83 (97.6%) students agreed that virtual OSCE teaching after the pandemic would be useful for preparation for medical school examinations.

The pre- and post- session confidence for all domains has been reported (Table 3) as well as the effect of the virtual OSCE teaching sessions on participants’ confidence which has also been reported with pre- and post-event medians along with statistical significance and effect sizes (Table 4). This indicated that there was a statistically significant improvement in participants’ confidence across all three OSCE domains as a result of attending the virtual OSCE sessions (*p* < 0.0001).

**Table 3:** The pre- and post- session confidence rating for each OSCE domain as rated by participants using a Likert scale

OSCE Domain	Not confident (Likert item 1)		Slightly confident (Likert item 2)		Somewhat confident (Likert item 3)		Confident (Likert item 4)		Very Confident (Likert item 5)	
	Pre	Post	Pre	Post	Pre	Post	Pre	Post	Pre	Post
History Taking	2 (2.4%)	0 (0%)	22 (25.9%)	2 (2.4%)	33 (38.8%)	33 (38.8%)	22 (25.9%)	55 (64.7%)	6 (7.1%)	17 (20%)
Communication	11 (12.9%)	0 (0%)	24 (28.2%)	2 (2.4%)	39 (45.9%)	16 (18.8%)	10 (11.2%)	55 (64.7%)	1 (1.2%)	12 (14.1%)
Data Analysis	11 (12.9%)	0 (0%)	27 (31.8%)	2 (2.4%)	36 (42.4%)	15 (17.6%)	10 (11.2%)	52 (61.2%)	3 (3.5%)	16 (18.8%)

**Table 4:** Effect of virtual OSCE teaching sessions on participants confidence with statistical significance and effect size (*n* = 85)

OSCE Domain	Pre-session confidence (median)	Post-session confidence (median)	*P*-value	Effect size (*r*)
History Taking	3	4	< 0.0001	1.373748
Communication	3	4	< 0.0001	1.662572
Data Analysis	3	4	< 0.0001	1.747822

Likert scale was used to determine pre and post-session confidence such that 1 = not confident and 5 = very confident

### Examiners

A total of 15 different examiners assisted in the virtual OSCE workshops with six examiners assisting in each workshop. The examiners consisted of 11 female and four male medical students in their clinical years across seven different UK-based medical schools. All examiners had previously undertaken an average of two formative OSCEs and one summative OSCE at their respective medical schools. Seven (46.7%) examiners had previous experience in delivering face-to-face OSCE teaching prior and all examiners had previously received either face-to-face OSCE teaching during medical school or small-scale virtual OSCE teaching prior to this workshop.

In comparison to the students that attended, 11 (73.3%) examiners agreed or strongly agreed that virtual OSCE teaching was as engaging as in-person OSCE teaching, and eight (53.3%) examiners agreed or strongly agreed that it was as interactive. Thirteen (86.7%) examiners agreed that virtual OSCE teaching after the pandemic would be useful for preparation for medical school examinations.

## Discussion

There has been a clear shift to online teaching as a result of the global COVID-19 pandemic, and virtual OSCE assessments organised by medical schools have been found to retain the same ability to assess communication skills, history-taking, clinical reasoning and formulating differential diagnoses and management plans as traditional face-to-face teaching [9]. Additional advantages over traditional in-person group teaching include the reduced travelling time, which could allow students to spend more time learning, whilst providing similar benefits to in-person teaching but in a more comfortable environment [14]. Virtual OSCE sessions also have the benefit of running larger and more regular sessions without physical constraints, such as booking rooms.

The overwhelmingly positive feedback received from students and the examiners indicated that this model of virtual mock OSCE delivery was likeable and feasible for both groups. As per the feedback, many students agreed or strongly agreed that the sessions were as interactive and engaging as in-person sessions, which is particularly important when considering the feasibility of shifting to an online format. Additionally, many agreed that online teaching would be useful for exam preparation, and this is further supported by the statistically significant improvement in confidence after these virtual sessions across all three domains: history-taking, communication and data-analysis (Table 3).

Moreover, studies running similar virtual OSCEs have shown that it is cost-efficient, useful and feasible in the current global situation with a potential role even in the post-pandemic phase [15]. As our OSCE workshops were all online, we were able to reach students on an international level, thus providing students from across the world with the opportunity to participate and access resources that they may have otherwise been unable to reach if the sessions were run in-person.

Another potential benefit of this format was the peer-led nature of the sessions, which could have helped examiners consolidate their own knowledge in a format that was perceived to be interactive, engaging and beneficial for OSCE preparation. This advantage has been demonstrated previously, where near-peer teaching has been shown to provide a mutually beneficial solution to all students involved, by consolidating knowledge for both tutors and tutees, whilst fostering confidence as educators [16].

Additionally, a significant drawback of the virtual format was that students were unable to practise examination skills and practical procedures online, both of which are essential skills required for summative OSCEs and clinical practice. It is possible that future studies could try to assess the feasibility of adopting a hybrid format or utilising virtual sessions to practice examinations and procedures. Moreover, further studies that directly compare academic outcomes between in-person and virtual OSCE teaching sessions are required to understand if there are any significant differences between the two teaching formats. Therefore, at present, it is not possible to comment on whether online teaching can fully replace clinical face-to-face teaching [14, 17].

Furthermore, although all five sessions were deemed to be successful, there were further limitations. One was in regards to hosting sessions for larger groups of students. This was due mainly to internet connection issues, which led to audio lag and difficulty accessing online resources during both the station and feedback sessions. Similar issues have been highlighted within the literature [10], with a systematic review of medical education during the COVID-19 pandemic showing that virtual teaching poses unique challenges, including reduced student engagement compared to in-person sessions [18]. We anticipate that replicating our workshops on a larger scale would amplify these issues and would require more time, preparation and administrative power to overcome. Other studies have further emphasised how planning and preparation is key to the smooth running of virtual OSCEs [10]. Further limitations were that the surveys, consisting of self-reported data, were completed by participants after the OSCE workshops to evaluate how they felt prior to the workshops. This is not a true pre- and post- survey as the participants only completed one survey after the intervention, which could have led to participants’ responses being impacted by recall bias, particularly with perceptions and opinions prior to the workshops. Additionally, there is an element of volunteer bias due the self-selective nature of recruiting participants. Moreover, the results are also affected by attrition bias as not every participant was able to answer the question comparing online teaching with in-person teaching as only 59 out of the 85 participants had previously attended in-person OSCE teaching, however, all participants answered the remaining questions. The aforementioned limitations, however, do decrease the ability to generalise the results from this study to a wider student population [19]. Moving forwards, overcoming these issues could be achieved by incorporating guidance from published literature on how to run virtual OSCEs by building on the lessons learnt from other organisations that have created similar online teaching programmes [11, 20]. Moreover, in future studies, in order to evaluate the value of virtual OSCE workshops, evaluation of students’ exam performances before and after would need to be undertaken. This would assess students’ learning and knowledge and whether these virtual OSCE sessions would significantly impact exam results.

## Conclusion

In summary, this national pilot study has demonstrated that virtual OSCE workshops are a feasible alternative to in-person OSCE teaching with the potential to continue to be beneficial beyond the pandemic. Of those who had also experienced in-person OSCE teaching, the peer-led virtual OSCE sessions were deemed, by the majority of students and examiners alike, to be interactive and engaging, with the majority of those students reporting an improvement in confidence in history-taking, communication and data interpretation skills. The majority of students and examiners also agreed that they would find virtual OSCE sessions to be beneficial when preparing for summative OSCEs even after the pandemic. More studies are required to assess the overall impact on student learning and to determine the value of virtual OSCE workshops on exam performance.

## Supplementary Information

**Additional file 1.**

**Additional file 2.**

**Additional file 3.**

## Data Availability

The raw data that support the findings of this study has been provided in a supplementary file in addition to this manuscript and is available by the corresponding author on reasonable request to any reader or scientist who wishes to use the data for non-commercial purposes and maintain the confidentiality.
