# Supplementary material for: Assessing the utility of virtual OSCE sessions as an educational tool: a national pilot study
Source: BMC Med Educ. 2022 Mar 15;22:178. doi: 10.1186/s12909-022-03248-3 (PMC8923093; doi:10.1186/s12909-022-03248-3)
Supplement: Supplementary file 2 — Additional file 2. [file 12909_2022_3248_MOESM2_ESM.docx]

Additional file 2

| Email |  |
| --- | --- |
| Name |  |
| Sex | - Male - Female - Prefer not to say |
| Which medical school did you go to? Please state other if you are not currently enrolled in a medical school in the UK. |  |
| What stage of your medical training are you in currently? |  |
| Have you previously received in person OSCE teaching? | - Yes - No |
| If yes to the previous question, to what extent would you agree with the following statements in comparison to in-person teaching   - Online OSCE teaching is as engaging - Online OSCE teaching is as interactive - Online OSCE teaching enables me to develop my clinical skills - Online OSCE teaching provides me with appropriate feedback of my performance | - Strongly Disagree - Disagree - Neutral - Agree - Strongly Agree |
| Please rate (out of 5) your confidence in the following topics BEFORE the session   - (Specialty): History Taking - (Specialty): Communication - (Specialty): Data Interpretation | - 1 - Not Confident - 2 - 3 - 4 - 5 – Very Confident |
| Please rate (out of 5) your confidence in the following topics AFTER the session   - (Specialty): History Taking - (Specialty): Communication - (Specialty): Data Interpretation | - 1 - Not Confident - 2 - 3 - 4 - 5 – Very Confident |
| To what extent do you feel having online OSCE sessions is sufficient for preparing you for in-person OSCE examinations? | - 1 – Not sufficient at all - 2 - 3 - 4 - 5 – Very sufficient |
| To what extent do you feel that the content in this session covers your curriculum for your medical school’s clinical exam   - (Specialty): History Taking - (Specialty): Communication - (Specialty): Data Interpretation | - 1 – Does not cover - 2 - 3 - 4 - 5 – Covers very well |
| Do you feel online-based OSCE teaching would be useful for your learning after the pandemic? | - Yes - No |
| What did you enjoy about this session? |  |
| What could be done to improve this session in the future? |  |
| Consent | - I consent to the data in this questionnaire being used for research and educational purposes. |
