# Supplementary material for: Assessing the utility of virtual OSCE sessions as an educational tool: a national pilot study
Source: BMC Med Educ. 2022 Mar 15;22:178. doi: 10.1186/s12909-022-03248-3 (PMC8923093; doi:10.1186/s12909-022-03248-3)
Supplement: Supplementary file 3 — Additional file 3. [file 12909_2022_3248_MOESM3_ESM.docx]

Additional file 3

| Email |  |
| --- | --- |
| Name |  |
| Sex | - Male - Female - Prefer not to say |
| Which medical school did you go to? Please state other if you are not currently enrolled in a medical school in the UK. |  |
| What stage of your medical training are you in currently? |  |
| Which OSCE workshop session(s) did you facilitate? | - Cardiology - Respiratory - Gastroenterology - Neurology - Endocrinology |
| Do you have any prior formal experience in delivering the following? | - In-person OSCE teaching - Teaching other medical students either virtually or face-to-face - Online teaching of medical-related content |
| How many formative OSCE examinations have you sat at medical school? |  |
| How many summative OSCE examinations have you sat at medical school? |  |
| Have you ever attended in-person OSCE teaching sessions throughout your time at medical school? | - Yes - No |
| Have you ever attended virtual OSCE teaching sessions throughout your time at medical school? | - Yes - No |
| To what extent would you agree with the following statements in comparison to in-person teaching   - Online OSCE teaching is as engaging - Online OSCE teaching is as interactive | - Strongly Disagree - Disagree - Neutral - Agree - Strongly Agree |
| In your opinion, which of the following posed the greatest challenge when facilitating this virtual OSCE workshop? | - Technical difficulties (e.g. utilising the breakout room function on Zoom or microphone/camera difficulties) - Running short of time - Engaging with the students - Tailoring the session to students at various stages in medical school |
| Do you feel online-based OSCE teaching would be useful for learning after the pandemic? | - Yes   No |
| What could be done to improve this session in the future? |  |
| Consent | - I consent to the data in this questionnaire being used for research and educational purposes. |
